# Neurobehavioral phenotype of autism spectrum disorder associated with germline heterozygous mutations in *PTEN*

**DOI:** 10.1038/s41398-019-0588-1

**Published:** 2019-10-08

**Authors:** Robyn M. Busch, Siddharth Srivastava, Olivia Hogue, Thomas W. Frazier, Patricia Klaas, Antonio Hardan, Julian A. Martinez-Agosto, Mustafa Sahin, Charis Eng, on behalf of the Developmental Synaptopathies Consortium, Simon K. Warfield, Benoit Scherrer, Kira Dies, Rajna Filip-Dhima, Amanda Gulsrud, Ellen Hanson, Jennifer M. Phillips

**Affiliations:** 1 Department of Neurology, Neurological Institute, Cleveland Clinic, Cleveland, OH USA; 2 Genomic Medicine Institute, Cleveland Clinic, Cleveland, OH USA; 3 Department of Neurology, Boston Children’s Hospital, Harvard Medical School, Boston, MA USA; 4 Harvard Medical School and Boston Children’s Hospital, Boston, MA USA; 5 Department of Quantitative Health Sciences, Cleveland Clinic, Cleveland, OH USA; 6 Autism Speaks, Cleveland, OH USA; 7 Pediatrics Institute, Cleveland Clinic, Cleveland, OH USA; 8 Psychiatry and Behavioral Sciences, Stanford University Medical Center, Stanford, CA USA; 9 Department of Human Genetics, University of California Los Angeles, Los Angeles, CA USA; 10 Taussig Cancer Institute, Cleveland Clinic, Cleveland, OH USA; 11 Department of Genetics and Genome Sciences, Case Western Reserve University, Cleveland, OH USA; 12 Department of Radiology, Boston Children’s Hospital, Boston, MA USA; 13 Department of Neurology, Boston Children’s Hospital, Boston, MA USA; 14 UCLA Semel Institute for Neuroscience & Human Behavior, David Geffen School of Medicine at UCLA, Los Angeles, CA USA; 15 Department of Developmental Medicine, Boston Children’s Hospital, Boston, MA USA; 16 Department of Psychiatry, Stanford University, Stanford, CA USA

**Keywords:** Autism spectrum disorders, Scientific community, Clinical genetics

## Abstract

Germline mutations in *PTEN*, the gene that encodes phosphatase and tensin homolog, have been identified in up to 20% of children with autism spectrum disorder (ASD) and macrocephaly and are associated with marked abnormalities in the white matter of the brain. This study sought to characterize the neurobehavioral phenotype of *PTEN*-ASD. Comprehensive neurobehavioral evaluations were conducted in 36 participants (ages 3–21 years) with *PTEN*-ASD and compared to two groups of controls: non-syndromic ASD with macrocephaly (Macro-ASD, *n* = 25) and those with *PTEN* mutations without ASD (*PTEN*-no ASD, *n* = 23). Linear regression analysis or Kruskal–Wallis tests were used to examine group differences on neurobehavioral measures (cognitive, behavioral, sensory, and adaptive functioning) and, for select measures, one-sample t-tests were used to compare group performance to healthy control norms. These analyses revealed a distinct neuropsychological profile associated with mutations in *PTEN* suggesting primary disruption of frontal lobe systems (i.e., attention, impulsivity, reaction time, processing speed, and motor coordination). Cognitive deficits in *PTEN*-ASD are more severe than those in *PTEN*-no ASD and extend to other areas of neurobehavioral function, specifically, adaptive behavior and sensory deficits. While core ASD symptoms are similar in *PTEN*-ASD and Macro-ASD, *PTEN*-ASD had lower clinical ratings of autism severity and showed more sensory abnormalities suggestive of less sensory responsiveness. Together, these results suggest that *PTEN*-ASD has a distinct neurobehavioral phenotype compared to idiopathic ASD that is likely to warrant special consideration for overall assessment and treatment.

## Introduction

Autism spectrum disorder (ASD) is a neurodevelopmental disorder marked by impairments in social communication as well as restricted, repetitive patterns of interests, behaviors, and activities^1^. ASD is highly heritable and may result from a number of genetic anomalies, including chromosomal copy number variation, single gene mutations, epigenetic changes, and complex inheritance^2–5^. Despite this knowledge, only 10–20% of ASD cases have an identified etiology^6^. As such, most research to date has been conducted on cases with “idiopathic” ASD samples with marked phenotypic and genetic heterogeneity. As a result, it has been difficult to identify therapeutic targets in ASD. One way to surmount this challenge is to study ASD through the lens of specific genetic causes. This approach may serve to distill the complexity and heterogeneity of idiopathic ASD and address the possibility that seemingly unrelated genetic disorders associated with ASD may converge on final common pathways^7,8^. Therefore, better understanding of mechanistic commonalities among distinct causes may shed light on the pathogenesis of ASD, opening the door to potential targeted treatments^9,10^.

*PTEN* hamartoma tumor syndrome (PHTS) is one example of a genetic disorder associated with a relatively high prevalence of ASD. PHTS is caused by germline mutations in *PTEN*, which encodes phosphatase and tensin homolog, resulting in a multitude of presentations that range from increased cancer risks to macrocephaly and neurodevelopmental impairment^11–13^. By some estimates, the prevalence of ASD in PHTS is around 22%^14^, and studies suggest that up to 20% of individuals with ASD with macrocephaly may have a pathogenic *PTEN* variant^15–18^.

Compared to other ASD groups (i.e., macrocephalic ASD, normocephalic ASD) and healthy controls, individuals with ASD and PHTS appear to have a specific pattern of increased and poorly-developed white matter with deficits in processing speed and working memory that extend beyond overall reductions in intellectual functioning^19^. While there is evidence to suggest that patients with PHTS without ASD also have a pattern of cognitive performance that implicates the involvement of frontal lobe systems^20^, these patient groups have never been directly compared across a comprehensive neuropsychological battery to differentiate *PTEN*-specific and ASD-specific deficits. Furthermore, we are unaware of any studies that have comprehensively examined behavioral phenotypes in these patient groups.

The goal of the current study was to comprehensively characterize, cross-sectionally, the neurobehavioral phenotype of *PTEN-*ASD in a large, prospective cohort and to examine phenotypic differences between these individuals and two groups of controls (i.e., ASD with macrocephaly but without *PTEN* mutation [Macro-ASD], *PTEN* mutation without ASD [*PTEN*-no ASD]). Based on preliminary data in independent samples^19,20^, we hypothesized that: (1) *PTEN*-ASD would have lower cognitive scores than the other patient groups, particularly on measures of intelligence, processing speed, working memory, and motor functioning; (2) *PTEN-*ASD would have comparable autism symptom levels to Macro-ASD, but lower intelligence (due to differences in processing speed and/or working memory) and adaptive function; and (3) *PTEN*-no ASD would demonstrate reduced performance on measures sensitive to frontal lobe functioning compared to healthy control norms.

## Materials and methods

### Participants

Participants in this study were recruited from four large tertiary medical centers (Cleveland Clinic, Boston Children’s Hospital, Stanford University Medical Center, and University of California, Los Angeles) as part of an IRB-approved, ongoing, multicenter prospective study designed to examine the natural history of autism and germline heterozygous *PTEN* mutations (clinicaltrials.gov: NCT02461446). All potential study participants were screened by a clinical psychologist with expertise in ASD to determine if they met DSM-5 diagnostic criteria for ASD. All potential participants also underwent genetic testing to determine the presence/absence of a mutation in *PTEN*. Individuals were included in the study if they met the following criteria: (1) age 3–21 years; (2) confirmed diagnosis of ASD (based on consensus of expert clinician evaluation and Autism Diagnostic Observation Schedule-2) and/or a confirmed heterozygous mutation in *PTEN*; (3) English as primary communicative language; and (4) completion of baseline neuropsychological evaluation. In addition, individuals with ASD, but without a *PTEN* mutation, had to have a occipitofrontal head circumference ≥98th percentile to be included in the study. Informed consent for study participation was obtained from adult participants and/or a parent or legal guardian. Assent was obtained from all participants age 7 years and older who were cognitively able to provide same.

A total of 84 participants met study inclusion criteria and were categorized into one of the following study groups based on clinical diagnosis and results of *PTEN* genotyping: *PTEN*-ASD (*n* = 36; ASD + *PTEN* mutation), Macro-ASD (*n* = 25; ASD with macrocephaly, no *PTEN* mutation) and *PTEN*-no ASD (*n* = 23; no ASD, *PTEN* mutation). Sample size for recruitment in the ongoing study was determined based on pilot data which showed effect sizes (Cohen’s *d*) of 1.5–2.5, when comparing *PTEN*-ASD and Macro-ASD subjects.

### *PTEN* Genotyping

Germline genomic DNA was extracted in the Genomic Medicine Institute’s Genomic Medicine Biorepository. PCR-based LightCycler mutation scanning and semi-automated PCR-based Sanger sequencing (ABI3730xl in Genomics Core Facility) of exons 1 through 9 and flanking intronic regions of genomic DNA was performed as per routine in the Eng lab since 1997 to reveal germline intragenic mutations in exons 1–9 and in splice sites. All novel variants were checked for presence and frequency in 350 ancestry-matched, sex-matched population controls (standard in Eng lab). PCR-based sequence analysis of the extended promoter region was also performed with Sanger sequencing to reveal promoter variants. All identified promoter variants were subjected to reporter assay as well as routine function interrogation. Multiplex ligation-dependent probe amplification was used to reveal large deletions and rearrangements.

### Measures

All study participants completed a neuropsychological assessment that included cognitive and behavioral measures administered for research purposes. The cognitive battery included age-appropriate measures of global cognitive ability, attention/impulsivity, working memory, processing speed, language, and visuospatial skills. Due to anticipated severe symptom and intellectual impairment in at least a subset of cases, executive functions and motor coordination were not directly assessed, but rather inferred from parent/guardian ratings on standardized questionnaires. Parents/guardians also completed a number of inventories designed to measure autism symptoms, sensory processing, behavioral difficulties, and adaptive functioning. The specific cognitive and behavioral measures used in this study are outlined in Supplementary Table 1. All measures were scored according to published test manuals using age- and/or sex-corrected norms as available/appropriate. Higher scores on the cognitive measures generally reflect better cognitive performance, with the exception of the indices and subscales of the continuous performance test (CPT) and the Behavior Rating Inventory of Executive Function (BRIEF). In contrast, higher scores on the autism, sensory, and behavioral measures are typically indicative of greater symptom severity/behavioral difficulties, with the exception of the Short Sensory Profile (SSP).

### Analyses

Baseline descriptive statistics stratified by diagnosis (*PTEN*-ASD, Macro-ASD, *PTEN*-no ASD) were calculated (Table 1). Statistics are presented as means with standard deviations for normally-distributed variables and medians with interquartile ranges for non-normal variables. Not all participants completed all study measures. Incomplete evaluations were largely due to the patient’s inability to tolerate testing or to time constraints. For those patients who were assessed, but who could not complete a particular measure due to low functional capacity, a score one point lower than the lowest possible score for that measure was assigned for purposes of data analyses. Assessments that were incomplete due to time constraints were considered missing at random.

**Table 1:** Demographic characteristics of study groups

	*PTEN*-ASD *n* = 36	Macro-ASD *n* = 25	*PTEN*-no ASD *n* = 23
*Age* ^a^	8.8 (5.1) Range 3.1–19.6	12.9 (4.9) Range 4.2–21.5	8.6 (4.1) Range 3.6–18.7
*Sex*			
Female	8 (22.2)	3 (12.0)	9 (39.1)
Male	28 (77.8)	22 (88.0)	14 (60.9)
*Race*			
White/Caucasian	29 (80.6)	14 (56.0)	17 (73.9)
Black/African American	1 (2.8)	1 (4.0)	0
Asian	0	5 (20.0)	2 (8.7)
Multiracial	4 (11.1)	5 (20.0)	3 (13.0)
Unknown/Not Reported	2 (5.6)	0	1 (4.4)
*Ethnicity*			
Hispanic	6 (16.6)	3 (12.0)	1 (4.4)
Not Hispanic	29 (80.6)	22 (88.0)	21 (91.3)
Unknown/Not reported	1 (2.9)	0	1 (4.4)

ASD = autism spectrum disorder

^a^Values presented as mean (standard deviation). All other values in table are presented as number (percentage)

Chi-square analyses were used to examine differences in the proportion of missense versus truncating mutation types between *PTEN*-ASD and *PTEN*-no ASD.

The study groups were compared on cognitive and behavioral measures using the *PTEN*-ASD group as a reference. For normally-distributed variables, linear regression analysis was used. Residuals by fitted value plots were examined to verify homoscedasticity. Kruskal–Wallis tests with the Dwass, Steel, Critchlow-Fligner pairwise comparison tests were used to compare the non-normal variables. Due to the significant age difference among groups, the effect of age on each cognitive and behavioral measure was tested using Pearson or Spearman’s correlations where appropriate. If age was significantly associated with the measure, it was included as a control variable. Exploratory multiple regression analyses also examined IQ as a function of both group and measures of frontal lobe functioning, including processing speed and working memory.

The Autism Diagnostic Observation Schedule-Second Edition (ADOS-2) was administered only to participants with ASD after diagnosis was confirmed by an expert clinical psychologist. *PTEN*-ASD and Macro-ASD were compared using the Wilcoxon rank-sum test.

One sample *t*-tests were used to compare mean performance of *PTEN*-no ASD to healthy control norms.

Given the exploratory nature of this study and relatively small sample sizes, we report effect sizes (Cohen’s *d*) in addition to *p*-values for all analyses rather than correcting for multiple comparisons. Effect sizes were interpreted as follows: small effect = 0.20, medium effect = 0.50, large effect = 0.80. Groups displayed homogeneity of variance for all comparisons. Analyses were two-sided and conducted using SAS Studio v. 3.3. SAS code is available upon request.

## Results

### Demographic characteristics

There were no significant differences in sex, race, or ethnicity among the three study groups. Macro-ASD was significantly older on average than both *PTEN*-ASD and *PTEN*-no ASD (*p* = 0.002) [Table 1].

### Genetic analyses

A summary of the germline *PTEN* variants observed in the study cohort is provided in Supplementary Table 2. There was a similar proportion of missense mutations (52.8% vs. 34.8%, *p* = 0.176) and truncating variants (44.4% vs. 60.9%, *p* = 0.218) in *PTEN*-ASD compared to *PTEN*-no ASD.

### Neurobehavioral profile of *PTEN*-ASD

**Fig. 1:**
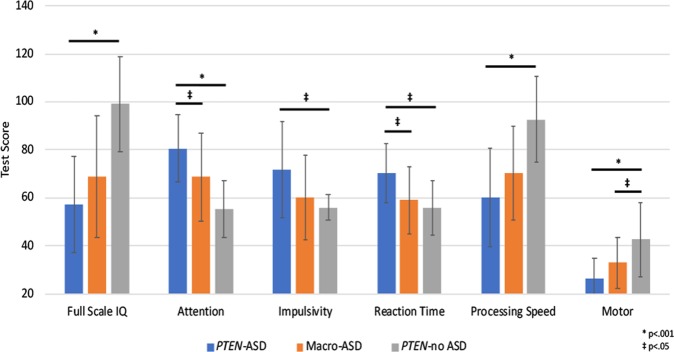
Group differences in cognitive and motor functioning. Higher scores indicate better cognitive performance for all domains, except attention, impulsivity, and reaction time. Full Scale IQ and processing speed are reported on a standard score scale (*M* = 100, SD = 15), attention, impulsivity, and reaction time are reported on a *t*-score scale (*M* = 50, SD = 10), and motor is reported as a raw score. The measures used to assess the noted cognitive domains are outlined in Supplementary Table 1. ASD, autism spectrum disorder

*PTEN*-ASD demonstrated poorer performance than *PTEN*-no ASD in every cognitive domain assessed, with effect sizes for most measures in the medium to large range (i.e., Cohen’s *d* range 0.41–2.21). Caregivers of children in the *PTEN*-ASD group also observed greater symptoms of behavioral and sensory dysfunction than caregivers of children in the *PTEN*-no ASD group, with similar range effects (*d* range 0.49–2.06) (Supplementary Table 3 and Fig. 1).

When compared to Macro-ASD, children with *PTEN*-ASD demonstrated greater cognitive impairments on measures of nonverbal intelligence (*d* = 0.65), attention (*d* *=* 0.73), reaction time (*d* = 0.85), and motor coordination (*d* = 0.69). While the threshold for statistical significance was not reached, medium to large effect sizes were also observed on measures of impulsivity (*d* = 0.61) and processing speed (*d* = 0.50) (Supplementary Table 4 and Fig. 1).

When controlling for working memory, the difference in FSIQ between *PTEN*-ASD and *PTEN*-no ASD decreased from 41.9 points to 6.3 points and the difference in FSIQ between *PTEN*-ASD and Macro-ASD decreased from 11.4 points to 5.5 points. Among subjects who completed the subtests that go into the Weschler Processing Speed Index, controlling for processing speed decreased the difference in FSIQ between *PTEN*-ASD and *PTEN*-no ASD from 46.7 points to 18.4 points and decreased the difference in FSIQ between *PTEN*-ASD and Macro-ASD from 19.3 points to 12.1 points.

**Fig. 2:**
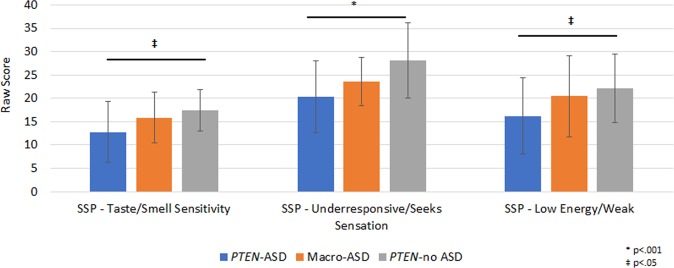
Group differences in sensory functioning. Higher scores indicate fewer sensory difficulties. Typical performance score ranges: Tactile Sensitivity 30–35, taste/smell sensitivity 15–20, under responsive/seeks sensation 27–35, low energy/weak 26–30. SSP, short sensory profile, ASD, autism spectrum disorder

*PTEN*-ASD had lower ADOS-2 Calibrated Severity Scores than Macro*-*ASD indicating less severe autism symptoms in this genetic sub-group (*d* = 0.56). While parents of both ASD groups noted less social responsiveness and more repetitive behavior than the *PTEN*-no ASD group, the two ASD groups did not significantly differ from each other on these scales (i.e., SRS and RBS-R) (Supplementary Tables 3 and 4). On caregiver-report questionnaires, *PTEN*-ASD were noted to have greater difficulties with sensory functioning than Macro-ASD (*d* = 0.62), particularly on the under-responsive/seeks sensation (*d* = 0.50), low energy/weak (*d* = 0.49), and taste/smell sensitivity (*d* = 0.50) scales (Supplementary Table 4 and Fig. 2). The two groups had similar symptoms on behavioral and adaptive behavior measures. (Supplementary Table 4).

Group performance on all cognitive and behavioral measures is summarized in Supplementary Tables 3 and 4, respectively, and a summary of observed neurobehavioral impairments by domain is provided in Table 2. It was necessary to control for age on CPT Commissions only.

**Table 2:** Summary of mean group performance on neurobehavioral measures^a^ by domain

	*PTEN*-ASD	Macro-ASD	*PTEN*-no ASD
Verbal intelligence	Severely impaired	Moderate impairment	High average
Nonverbal intelligence	Severely impaired	Mildly impaired	Average
Attention	Severely impaired	Mildly impaired	Average
Working memory	Moderately impaired	Moderately impaired	Low average to average
Impulsivity	Mildly impaired	Low average	Average
Processing speed	Moderately impaired	Mildly imapired	Average
Reaction time	Mildly impaired	Low average	Average
Executive functions	Mildly impaired	Mildly impaired	Low average
Expressive language	Moderately impaired	Mildly impaired	Average
Receptive language	Moderately impaired	Mildly impaired	High average
Visuospatial	Moderately impaired	Mildly impaired	Average
Motor	Severely impaired	Moderately impaired	Mildly impaired
Sensory functioning	Severely impaired	Severely impaired	Moderately impaired
Problem behavior	Moderately impaired	Moderately impaired	Average

*ASD* autism spectrum disorder

^a^Categories based on group mean standard scores. Severe impairment (<60), moderate impairment (60–69), mild impairment (70–79), low average (80–89), average (90–109), high average (110–119)

### Neurobehavioral profile of *PTEN-*no ASD

**Fig. 3:**
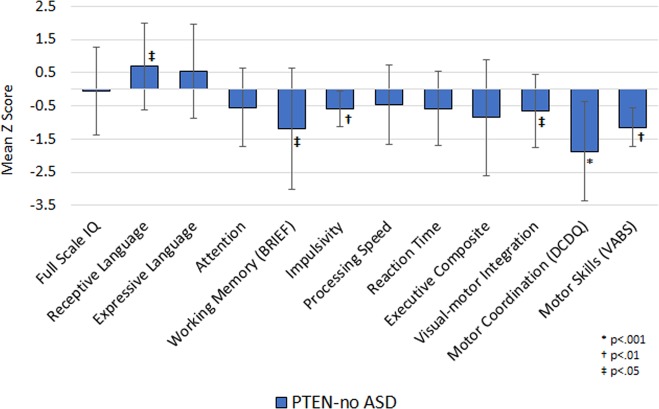
Cognitive performance in *PTEN*-ASD compared to healthy control norms. Upgoing bars indicate performances *higher* than normative mean z-score of 0, and downgoing bars indicate performances *lower* than normative mean z-score of 0. BRIEF, Behavior Rating Inventory of Executive Function, DCDQ, Developmental Coordination Disorder Questionnaire, VABS, Vineland Adaptive Behavior Scale, ASD, autism spectrum disorder

*PTEN*-no ASD did not significantly differ from established healthy control norms on measures of global cognitive ability. However, they had reduced mean scores compared to healthy controls on measures of subjective working memory (*p* = 0.011, *d* = 0.81), impulsivity (*p* = 0.002, *d* = 0.75), visuomotor integration (*p* = 0.012, *d* = 0.62), and motor coordination (*p* < 0.001, *d* = 1.48). In contrast, their scores on a measure of receptive vocabulary were significantly higher than established healthy controls norms (*p* = 0.021, *d* = 0.60) (Fig. 3).

On adaptive functioning measurements, *PTEN*-no ASD had reduced scores on the Motor Skills (*p* < 0.001, *d* = 0.95) and Internalizing (*p* = 0.035, *d* = 0.57) subscales of the Vineland Adaptive Behavior Inventory—Second Edition compared to healthy control norms. Caregivers of children in the *PTEN*-no ASD group also reported more repetitive behaviors on the Repetitive Behavior Scale compared to healthy control norms (*p* = 0.004, *d* = 0.99). Interestingly, the mean scores of *PTEN*-no ASD were below normative expectation on the total score of the Short Sensory Profile suggesting greater sensory difficulties. Examination of the subtest scores revealed the most notable difficulties with low energy/weakness and auditory filtering.

## Discussion

We have identified a distinct neuropsychological profile associated with mutations in *PTEN* suggesting primary disruption of frontal lobe systems^21,22^. Specifically, individuals with *PTEN* mutations showed reduced performance on measures of attention, impulsivity, reaction time, processing speed, and motor coordination, consistent with our pilot findings in independent patient cohorts^19,20^. This pattern of cognitive deficits may be related to neuroanatomical abnormalities. *PTEN* is a dual-specificity phosphatase that is a major inhibitor of the PI3K-AKT pathway^23–25^ with downstream effects on mTOR signaling, which helps regulate neural cell growth and proliferation^26^. Germline mutations in *PTEN* are associated with macrocephaly, with brain MRI showing markedly larger brain volumes as well as increased white matter hypointensities compared to other patient groups and healthy controls^19,27,28^. Even after controlling for total intracranial volume, volumetric differences remain between individuals with *PTEN* mutations and healthy controls in total white matter as well as in a number of specific brain regions, including the corpus callosum and cerebellum. While cortical thickness appears to vary across brain regions, our recent findings suggest reduced cortical thickness in the frontal regions of individuals with *PTEN* mutations^19,28^, which may account for our cognitive observations.

*Pten*^*m3m4*^ murine model, a constitutional *Pten* knock-in model with autism-like phenotypes, demonstrate upregulated expression of genes implicated in myelination, including myelin basic protein and myelin proteolipid protein, possibly contributing to increased volumes of white matter structures^19^. Oligodendrocyte development relies on precise transcriptional control of genes involved in structural support of myelin^29^, and deregulation of this tightly controlled process may lead to white matter abnormalities. While behavioral studies in *Pten*^*m3m4*^ mice have focused primarily on autism phenotypes (e.g., social interaction, repetitive behavior, anxiety)^16^, these mice do not appear to have global cognitive dysfunction and there is some evidence to suggest motor coordination deficits and social difficulties as observed in humans with *PTEN* mutations^16^. To our knowledge, attention, working memory and other more “frontal” type tasks have not yet been assessed in rodent models of Pten. Further research in this regard will be needed to validate our findings. It is also important to note that we cannot rule out the possibility that cerebellar dysfunction may contribute to this pattern given the known role of the cerebellum in many aspects of motor and cognitive functioning^30–32^.

While both *PTEN* groups showed cognitive patterns suggesting primary involvement of frontal lobe systems, *PTEN*-ASD had deficits that were more severe than *PTEN*-no ASD and extended to other areas of neurobehavioral function. Specifically, cognitive deficits in *PTEN*-no ASD tended to be mild and largely restricted to measures sensitive to frontal lobe function, whereas *PTEN*-ASD showed frontal deficits in the moderate to severe range accompanied by moderate to severe impairments in intellectual functioning and moderate deficits in both expressive and receptive language suggesting involvement of more widespread brain regions (Table 2). Interestingly, both *PTEN* groups reported deficits in sensory functioning, with the most notable problems on the under-responsive/seeks sensation, low energy/weak and taste/smell sensitivity subscales of the Short Sensory Profile (Fig. 3 and Supplementary Tables 3 and 4). The more severe neurobehavioral phenotype observed in *PTEN*-ASD may be related to more extensive brain abnormalities compared to *PTEN*-no ASD. While we are unaware of any studies that have directly compared neuroimaging characteristics of these two patient groups, anectodally *PTEN*-ASD appears to have greater white matter volume than *PTEN*-no ASD. This hypothesis remains to be confirmed in future studies with age-matched samples.

Similarly, while both ASD groups demonstrated rather global cognitive impairments, *PTEN*-ASD showed more severe impairments than those with Macro-ASD, particularly on measures associated with frontal systems dysfunction (e.g., attention, reaction time, motor function) (Supplementary Table 4). These findings are consistent with prior research in a small independent cohort suggesting reduced cognitive functioning in *PTEN-*ASD versus idiopathic ASD^19^ and likely reflect specific brain alterations including white matter abnormalities associated with *PTEN* mutations as previously discussed. In contrast, core ASD symptoms in *PTEN-*ASD appear to be similar to those seen in idiopathic ASD. *PTEN-*ASD and Macro-ASD reported similarities in social responsiveness and repetitive behavior (as assessed by the SRS and RBS, respectively); however, *PTEN*-ASD had lower clinical ratings of autism severity on the ADOS-2. This may reflect the passive/slower presentation of *PTEN-*ASD rather than a true reduction in autism severity. Indeed, caregiver responses on a sensory functioning measure suggest that *PTEN*-ASD often presents with low energy/weakness and under responsiveness. These findings are quite consistent with parent reports as well as our qualitative observations in the clinic.

Importantly, these study results suggest that the slow processing speed/reaction times, attention/working-memory difficulties, and motor deficits observed in individuals with *PTEN* mutations likely impact other aspects of their test performance. While mean FSIQ in *PTEN*-no ASD was within the average range (i.e., standard score of 99.2), scores on an untimed measure of receptive language (Peabody Picture Vocabulary Test) known to be highly correlated with FSIQ were substantially higher (i.e., standard score 110.4, high average range). This suggests that FSIQ, which has attention/working memory, speed, and motor components, may underestimate general cognitive ability in *PTEN* patients. Indeed, our data would support this conclusion as differences in FSIQ observed between groups were much less apparent after adjusting for working memory and processing speed.

The rather consistent pattern of neurobehavioral deficits observed in individuals with *PTEN* muations is likely to have important clinical and educational implications, particularly for those diagnosed in childhood, and help contribute to our understanding of the underlying pathophysiology of autism symptoms and/or cognitive deficits in those with *PTEN* mutations. As such, comprehensive neuropsychological evaluation should be considered in children, adolescents, and young adults with *PTEN* mutations to thoroughly assess social and behavioral functioning as well as to identify cognitive strengths and weaknesses. Clinicians, and researchers, should also be sure to assess attention, speed and motor functions as part of any cognitive evaluation and to take those results into account when interpreting other test data and making clinical and educational recommendations. These type of assessments can inform tailored recommendations for academic accommodations and targeted interventions to optimize cognitive performance, adaptive functioning, and educational outcomes. For example, individuals with slow processing speed and/or attention/working memory difficulties may require information to be presented at a more deliberate pace and may benefit from brief delays in conversation to permit the formulation of their response. These individuals may also require academic accommodations to permit more time for assignments and tests, and educational expectations should be tempered. Tests that rely on motor coordination and speed should be avoided given the marked motor deficits associated with mutations in *PTEN*.

Taken together, results of this study suggest that children with *PTEN*-ASD have a distinct neurobehavioral phenotype and, as such, are likely to vary in several aspects of clinical presentation from patients with idiopathic ASD. Clinicians should make note of these cognitive and behavioral differences and be wary not to under-identify autism in individuals with *PTEN* mutations, as clinical observations may confound a relatively passive/slower processing phenotype with autism symptom severity. As in all individuals with autism, early identification is key to accessing evidence-based early interventions and maximizing developmental trajectories^33–35^. Comprehensive neuropsychological evaluation is recommended for the identification of cognitive strengths as well as weaknesses that may require remediation and/or accommodation. Similarly, the marked deficits in motor and sensory functioning that often accompany *PTEN*-ASD are likely to warrant thorough occupational and physical therapy evaluations for most patients. In contrast to idiopathic ASD, our results suggest that individuals with *PTEN*-ASD may require less in terms of assessment and/or intervention planning for challenging behavior and more with regard to cognitive rehabilitation/treatment. Ultimately, it is important for clinicians, parents, caregivers, and teachers to be made aware of these important phenotypic differences in order to obtain appropriate interventions and maximize day-to-day functioning.

There are a few limitations of the current study that deserve mention. There was a small subset of children in the study who were too low functioning to complete all cognitive measures. Rather than limit our dataset only to those with complete data and risk the potential of overestimating cognitive ability in this cohort, we assigned one lower than the lowest possible score for tasks that were attempted, but could not be completed. Further, while sample sizes in this cohort are among the highest reported to date, analyses for some measures were underpowered to detect medium effect sizes. In order to address this issue, we calculated effect sizes for each of the comparisons rather than rely solely on traditional *p*-values. It is also important to note that the Macro-ASD group was older than the two *PTEN* groups and, while not statistically significant, had a smaller proportion of females. Expanded recruitment in the overarching study is planned to achieve better demographic balance among groups and increase sample size, thereby improving power to detect smaller effects. Finally, while these cross-sectional data highlight some distinct neurobehavioral characteristics associated with *PTEN*-ASD, they give only a snapshot of functioning at a single time point in childhood/adolescence. Thus, efforts are currently underway to follow these children longitudinally and to extend recruitment to a broader age range (18 months to 45 years) in order to examine the evolution of neurobehavioral functioning in *PTEN*-ASD throughout development and into adulthood. Finally, future research will seek to better understand the mechanisms that drive the neurobehavioral differences we have observed. Our future work will examine relationships between neurobehavioral variables and underlying molecular and neurophysiological systems as well as neuroimaging measures. Equipped with this information, we hope to build a comprehensive longitudinal cross-level model that builds key links from *PTEN* mutations to neurobehavioral outcomes.

## Supplementary information

Supplemental Material Legend

Supplemental Table 1

Supplemental Table 2

Supplemental Table 3

Supplemental Table 4
