# Supplementary material for: Neurobehavioral phenotype of autism spectrum disorder associated with germline heterozygous mutations in PTEN
Source: Transl Psychiatry. 2019 Oct 8;9:253. doi: 10.1038/s41398-019-0588-1 (PMC6783427; doi:10.1038/s41398-019-0588-1)
Supplement: Supplementary file 1 — Supplemental Material Legend [file 41398_2019_588_MOESM1_ESM.docx]

**Supplemental Material**

**Legends**

**Supplemental Table 1.** Cognitive and Behavioral Measures

**Supplemental Table 2.** Germline *PTEN* Nucleotide Variants

**Supplemental Table 3.** Group Differences on Cognitive and Behavioral Measures Between *PTEN*-ASD and *PTEN*-no ASD

**Supplemental Table 4.** Group Differences on Cognitive and Behavioral Measures Between *PTEN*-ASD and Macro-ASD
