# Supplementary material for: Neurobehavioral phenotype of autism spectrum disorder associated with germline heterozygous mutations in PTEN
Source: Transl Psychiatry. 2019 Oct 8;9:253. doi: 10.1038/s41398-019-0588-1 (PMC6783427; doi:10.1038/s41398-019-0588-1)
Supplement: Supplementary file 2 — Supplemental Table 1 [file 41398_2019_588_MOESM2_ESM.docx]

| **Supplemental Table 1. Cognitive and Behavioral Measures2.** | | | |
| --- | --- | --- | --- |
| **Domain** | **Measure** | **Abbreviation** | **Scales** |
| **Global Ability** | Stanford-Binet Intelligence Scales – Fifth Edition (34) | SBIC | Full Scale IQ (FSIQ), Verbal IQ (VIQ), Non-Verbal IQ (NVIQ) |
| **Attention** | Conners’ Continuous Performance Test (35) | CPT | Omissions^a^ |
| **Impulsivity** | Conners’ Continuous Performance Test (35) | CPT | Commissions^a^ |
| **Working Memory** | Stanford-Binet Intelligence Scales – Fifth Edition (34) | SB | Working Memory |
| **Processing Speed** | Wechsler Preschool and Primary Scale of Intelligence – Fourth Edition, Wechsler Intelligence Scale for Children – Fifth Edition, or Wechsler Adult Intelligence Scale – Fourth Edition (36) | WPPSI, WISC, or WAIS | Processing Speed Index (PSI) |
|  | Connors’ Continuous Performance Test – Third Edition or Connors’ Kiddie Continuous Performance Test - 2^nd^ Edition (35) | CPT | Hit Reaction Time^a^ |
| **Language** | Peabody Picture Vocabulary Test – Fourth Edition (37) | PPVT | Total Score |
|  | Expressive Vocabulary Test – Fourth Edition (38) | EVT | Total Score |
| **Executive Functioning** | Behavior Rating Inventory of Executive Function (39) ^c^ | BRIEF | Global Executive Composite^a^  Index Scores^a^  Subscale Scores^a^ |
| **Visuospatial** | Beery-Buktenica Developmental Test of Visual-Motor Integration – Sixth Edition (40) | VMI | Total Score |
| **Motor** | Developmental Coordination Disorder Questionnaire (41)^c^ | DCDQ | Total Score |
| **Autism Symptoms** | Autism Diagnostic Observation Schedule – Second Edition (42) | ADOS-2 | Calibrated Severity Score |
|  | Social Responsiveness Scale – Second Edition (43)^c^ | SRS | Total Score |
|  | Repetitive Behavior Scale – Revised (44)^c^ | RBS | Total Score |
| **Behavioral Functioning** | Child Behavior Checklist (45)^c^ | CBCL | Externalizing Scale  Internalizing Scale  Total Problems Score |
| **Sensory Functioning** | Short Sensory Profile (46)^c^ | SSP | Factor Scores^b^ |
| **Adaptive Functioning** | Vineland Adaptive Behavior Scales – Second Edition (47)^c^ | VABS-2 | Clinical Scales  Internalizing Scale  Externalizing Scale |

IQ=intelligence quotient

^a^ Higher scores indicate poorer cognitive performance

^b^ Higher scores indicate lower symptom severity

^c^ Parent/caregiver report measure
