# Supplementary material for: Neurobehavioral phenotype of autism spectrum disorder associated with germline heterozygous mutations in PTEN
Source: Transl Psychiatry. 2019 Oct 8;9:253. doi: 10.1038/s41398-019-0588-1 (PMC6783427; doi:10.1038/s41398-019-0588-1)
Supplement: Supplementary file 3 — Supplemental Table 2 [file 41398_2019_588_MOESM3_ESM.docx]

**Supplemental Table 2. *PTEN* Nucleotide Variants**

| ***PTEN-ASD*** | | ***PTEN*-No ASD** | | **Macro-ASD** | |  |
| --- | --- | --- | --- | --- | --- | --- |
| **ID No.** | | **ID No.** | | **ID No.** |  |  |
| 01-001 | c.556_557delCT (p.Leu186fs*3) | 01-004 | c.3G>T (p. Met1Ile) | 01-010 | Mutation Negative |  |
|  | c.1026+32T>G^a^ | 01-007 | c.494G>T (p.Gly165Val) | 01-013 | Mutation Negative |  |
| 01-002 | c.3G>T (p.Met1Ile) | 01-009 | c.388C>T (p.Arg130*) | 01-028 | Mutation Negative |  |
|  | c.1026+32T>G^a^ |  | c.1026+32T>G^a^ | 02-004 | c.1026+32T>G^a^ |  |
| 01-003 | c.3G>T (p.Met1Ile) | 01-011 | c.511C>T (p.Gln171*) | 02-009 | c.1026+32T>G^a^ |  |
| 01-005 | c.388C>T (p.Arg130*) | 01-012 | c.97_99delATT (p.Ile33del) | 02-012 | c.1026+32T>G^a^ | |
|  | c.1026+32T>G^a^ |  | c.1026+32T>G^a^ | 03-001 | c.1026+32T>G^a^ |  |
|  | c.802-51_802-14del38 | 01-015 | c.740dupA (p.Pro248Thrfs*5) | 03-002 | c.1026+32T>G^a^ |  |
| 01-006 | c.1003C>T (p.Arg335*) |  | c.1026+32T>G^a^ | 03-003 | c.1026+32T>G^a^ |  |
| 01-008 | c.494G>T (p.Gly165Val) | 01-016 | c.388C>T (p.Arg130Gly) | 03-004 | c.1026+32T>G^a^ |  |
| 01-014 | c.697C>T (p.Arg233*) | 01-022 | c.886delT (Cys296Varfs*11) | 03-005 | c.1026+32T>G^a^ |  |
| 01-018 | c.287C>A (p. Pro96Gln) |  | c.1026+32T>G |  | c.-903G>A^a^ |  |
|  | c.1026+32T>G^a^ | 01-023 | c.511C>T (p.Gln171*) | 03-006 | Mutation Negative |  |
| 01-021 | c.1061C>A (p.Pro354Gln) | 01-027 | c.204C>A (p. Tyr68*) | 03-008 | c.1026+32T>G^a^ |  |
| 01-024 | c.277C>T (p. His93Tyr) | 01-034 | c.210-2A>G (exon 4 skipping; | 03-009 | c.1026+32T>G^a^ |  |
|  | c.1026+32T>G^a^ |  | p.Ala72Thrfs*5) | 03-010 | c.-903G>A^a^ |  |
| 01-025 | c.395G>A (p.Gly132Asp) | 01-036 | c.955_958delACTT (p.Thr319Terfs) | 03-012 | Mutation Negative |  |
|  | c.1026+32T>G^a^ |  | c.1026+32T>G^a^ | 03-013 | Mutation Negative |  |
| 01-026 | c.379G>T (p.Gly127Ter) | 01-040 | c.277C>G (His93Asp) | 03-015 | Mutation Negative |  |
| 01-029 | c.80-3C>G (intronic - ?no effect) |  | c.1026+32T>G^a^ | 03-017 | c.1026+32T>G^a^ |  |
|  | c.-903G>A^a^ | 01-043 | c.1003C>T (Arg335Ter) | 04-006 | c.1026+32T>G^a^ |  |
|  | c.634+4A>T (p.Gly165llefs[*](https://onlinelibrary.wiley.com/doi/full/10.1002/humu.23288#humu23288-tbl1-note-0001)9) | 01-045 | c.703delG (p.Glu235Lysfs*21) | 04-008 | Mutation Negative |  |
| 01-031 | c.517C>T (p. Arg173Cys) |  | c.1026+32T>G | 04-009 | c.1026+32T>G^a^ |  |
| 01-032 | c.385G>A (p.Gly129Arg) | 01-046 | c.947delT (p.Leu316Glnfs) | 04-014 | c.1026+32T>G^a^ |  |
|  | c.1026+32T>G^a^ | 01-048 | c.203A>G (Tyr68Cys) | 04-015 | Mutation Negative |  |
| 01-033 | c.103A>G (p.Met35Val) | 02-002 | c.737C>T (p.Pro246Leu) | 04-017 | Mutation Negative |  |
| 01-035 | c.3G>A (p.Met1Ile) | 02-006 | c.494G>T (p.Gly165Val) |  |  |  |
|  | c.1026+32T>G^a^ | 02-008 | c.521A>G (p.Tyr174Cys) |  |  |  |
| 01-038 | c.944_945delAT (p.Tyr315Serfs) |  | c.1026+32T>G^a^ |  |  |  |
|  | c.1026+32T>G^a^ | 03-011 | Del promoter through exon 5 |  |  |  |
| 01-039 | c.277C>G (p.His93Asp) | 03-014 | c.632dupG (p.Cys211Trpfs) |  |  |  |
|  | c.1026+32T>G^a^ |  | c.1026+32T>G^a^ |  |  |  |
| 01-041 | c.386_387delinsTT (p.Gly129Val) | 03-018 | c.912delC(Cys304Ter) |  |  |  |
|  | c.1026+32T>G^a^ |  | c.1026+32T>G^a^ |  |  |  |
| 01-042 | c.1003C>T (p.Arg335*) |  |  |  |  |  |
| 01-044 | c.464T>G(p.Tyr155Cys) |  |  |  |  |  |
|  | c.1026+32T>G^a^ |  |  |  |  |  |
| 01-047 | c.-975G>A |  |  |  |  |  |
|  | c.1026+32T>G^a^ |  |  |  |  |  |
| 01-049 | c.1212A>T |  |  |  |  |  |
|  | c.1026+32T>G^a^  c.132C>T^a^ |  |  |  |  |  |
| 02-001 | c.389G>A (p. Arg130Gln) |  |  |  |  |  |
| 02-005 | c.-1084C>T^a^ |  |  |  |  |  |
|  | c.728delT (p.Phe243fs*13) |  |  |  |  |  |
|  | c.1026+32T>G^a^ |  |  |  |  |  |
| 02-011 | c.475A>G (p.Argl59Gly) |  |  |  |  |  |
| 02-014 | c.388C>T (p.Arg130*) |  |  |  |  |  |
| 02-017 | c.610C>G(p.Pro204Ala) |  |  |  |  |  |
|  |  |  |  |  |  |  |
|  |  |  |  |  |  |  |
|  |  |  |  |  |  |  |
| 03-007 | c.165-2A>G (intron 2, p.Arg55Ser+p.Phe56_Leu70del) |  |  |  |  |  |
|  | c.1026+32T>G^a^ |  |  |  |  |  |
| 04-001 | c.683delA (p.Lys228fs*23) |  |  |  |  |  |
| 04-002 | c.395G>A (p. Gly132Asp) |  |  |  |  |  |
| 04-003 | c.388C>T (p.Arg130*) |  |  |  |  |  |
|  | c.1026+32T>G^a^ |  |  |  |  |  |
| 04-005 | c.-729C>T (promoter) |  |  |  |  |  |
|  | c.1026+32T>G^a^ |  |  |  |  |  |
| 04-011 | c.389G>A (p.Arg130Gln) |  |  |  |  |  |
|  | c.1026+32T>G^a^ |  |  |  |  |  |
| 04-012 | c.999dupC (p.Asn334GInfs*9) |  |  |  |  |  |

ASD=autism spectrum disorder

^a^ Benign polymorphisms
