# Supplementary material for: Neurobehavioral phenotype of autism spectrum disorder associated with germline heterozygous mutations in PTEN
Source: Transl Psychiatry. 2019 Oct 8;9:253. doi: 10.1038/s41398-019-0588-1 (PMC6783427; doi:10.1038/s41398-019-0588-1)
Supplement: Supplementary file 4 — Supplemental Table 3 [file 41398_2019_588_MOESM4_ESM.docx]

| **Supplemental Table 3. Group Differences on Cognitive & Behavioral Measures Between *PTEN*-ASD**  **and *PTEN*-no ASD** | | | | | |
| --- | --- | --- | --- | --- | --- |
|  | ***PTEN-ASD***  **n = 36** | ***PTEN*-no ASD**  **n = 23** | **p** | **Cohen’s d** |  |
| **Global Cognitive Ability** | | | | |  |
| **Stanford Binet Intelligence Scales** |  |  |  |  |  |
| **Full Scale IQ**  standard score, mean(SD) | 57.3 (20.1)  n = 33 | 99.2 (19.9)  n = 22 | **<.001** | **2.09** |  |
| **Verbal IQ**  standard score, mean(SD) | 58.2 (21.7)  n = 33 | 100.7 (20.2)  n = 22 | **<.001** | **2.03** |  |
| **Nonverbal IQ**  standard score, mean(SD) | 58.1 (18.9)  n = 33 | 99.5 (18.5)  n = 22 | **<.001** | **2.21** |  |
| **Attention / Impulsivity** |  |  |  |  |  |
| **Continuous Performance Test** |  |  |  |  |  |
| **Omissions**^a^  T-score, mean (SD) | 80.7 (13.8)  n = 18 | 55.5 (11.8)  n = 13 | **<.001** | **2.05** |  |
| **Commissions**  T-score, mean (SD)^‡,a^ | 69.5 (4.0)  n = 18 | 54.7 (4.6)  n =13 | **.016** | **1.07** |  |
| **Working Memory** |  |  |  |  |  |
| **Stanford Binet Intelligence Scales** |  |  |  |  |  |
| **Working Memory**  standard score, mean (SD) | 62.5 (22.0)  n = 33 | 102.5 (20.0)  n = 22 | **<.001** | **1.90** |  |
| **Processing Speed** |  |  |  |  |  |
| **Wechsler Processing Speed Index**  Standard score, mean (SD) | 60.3 (20.5)  n = 23 | 92.8 (18.1)  n = 19 | **<.001** | **1.68** |  |
| **Continuous Performance Test** |  |  |  |  |  |
| **Hit Reaction Time**  T-score, mean (SD)^a^ | 70.3 (12.2)  n = 10 | 55.8 (11.3)  n = 13 | **.010** | **1.23** |  |
| **Executive Functions** |  |  |  |  |  |
| **Behavior Rating Inventory of Executive Function** | | | |  |  |
| **Global Executive Composite**  T-score, mean (SD)^a^ | 66.4 (11.0)  n = 27 | 58.6 (17.5)  n = 18 | .051 | **0.53** |  |
| **Behavioral Regulation Index**  T-score, mean (SD)^a^ | 62.8 (13.0)  n = 21 | 54.0 (16.8)  n = 15 | .071 | **0.59** |  |
| **Inhibit**  T-score, mean (SD)^a^ | 63.9 (13.4)  n = 28 | 52.7 (13.1)  n = 19 | **.008** | **0.84** |  |
| **Shift**  T-score, mean (SD)^a^ | 62.1 (12.2)  n = 28 | 55.3 (15.7)  n = 19 | .095 | 0.48 |  |
| **Emotional Control**  T-score, mean (SD)^a^ | 58.9 (12.9)  n = 28 | 52.2 (14.6)  n = 19 | .098 | 0.49 |  |
| **Metacognition Index**  T-score, mean (SD)^a^ | 65.0 (12.1)  n = 22 | 58.3 (16.9)  n = 14 | .121 | 0.46 |  |
| **Initiate**  T score, mean (SD)^a^ | 64.6 (13.4)  n = 21 | 58.6 (15.9)  n = 15 | .181 | 0.41 |  |
| **Working Memory**  T-score, mean (SD)^a^ | 68.9 (15.0)  n = 29 | 61.9 (18.3)  n = 19 | .122 | 0.42 |  |
| **Plan/Organize**  T-score, mean (SD)^a^ | 65.6 (15.9)  n = 28 | 57.8 (17.2)  n = 18 | .083 | 0.47 |  |
| **Organization of Materials**  T-score, mean (SD)^a^ | 54.4 (11.3)  n = 21 | 52.9 (13.6)  n = 15 | .702 | 0.12 |  |
| **Monitor**  T-score, med (IQR)^a^ | 71 (58-76)  n = 20 | 51 (40-64)  n = 15 | **<.015** | **1.06** |  |
| **Language** |  |  |  |  |  |
| **Peabody Picture Vocabulary Test**^‡^  standard score, mean (SD) | 61.1 (32.6)  n = 34 | 110.4 (19.5)  n = 22 | **<.001** | **1.84** |  |
| **Expressive Vocabulary Test**  standard score, mean (SD) | 60.7 (30.7)  n = 33 | 107.9 (21.3)  n = 22 | **<.001** | **1.79** |  |
| **Visuospatial** |  |  |  |  |  |
| **Visual-Motor Integration**  standard score, mean (SD) | 63.2 (20.2)  n = 33 | 90.1 (16.7)  n = 22 | **<.001** | **1.45** |  |
| **Motor** |  |  |  |  |  |
| **Developmental Coordination Disorder Questionnaire**  raw score, mean (SD)^b^ | 26.3 (8.7)  n = 32 | 42.6 (15.3)  n = 20 | **<.001** | **1.31** |  |
| **Autism Symptoms** |  |  |  |  |  |
| **Social Responsiveness Scale**  T-score, med (IQR) | 76.5 (70-83)  n = 34 | 50 (44-68)  n = 18 | **.001** | **1.34** |  |
| **Repetitive Behavior Scale**  raw score, med (IQR)^c^ | 21 (12-48)  n = 33 | 3 (1-15)  n = 21 | **<.001** | **1.45** |  |
| **Sensory Functioning** |  |  |  |  |  |
| **Short Sensory Profile** |  |  |  |  |  |
| **Tactile Sensitivity**  raw score, med (IQR)^d^ | 27 (22-31)  n = 33 | 32 (30-35)  n = 21 | **.002** | **1.10** |  |
| **Taste/Smell Sensitivity**  raw score, med (IQR)^d^ | 13 (7-20)  n = 33 | 19 (18-20)  n = 21 | **.03** | **0.83** |  |
| **Movement Sensitivity**  raw score, med (IQR)^d^ | 12 (9-15)  n = 33 | 14 (12-15)  n = 21 | .213 | **0.57** |  |
| **Under-responsive/Seeks Sensation**  raw score, mean (SD)^d^ | 20.3 (7.7)  n = 33 | 28.1 (8.0)  n = 21 | **<.001** | **0.99** |  |
| **Auditory Filtering**  raw score, mean (SD)^d^ | 18.0 (5.1)  n = 33 | 22.5 (6.0)  n = 21 | **.002** | **0.82** |  |
| **Low Energy/Weak**  raw score, med (IQR)^d^ | 15 (8-23)  n = 33 | 23 (16-29)  n = 21 | **.027** | **0.77** |  |
| **Visual/Auditory Sensitivity**  raw score, mean (SD)^d^ | 16.2 (4.8)  n = 33 | 19.0 (6.4)  n = 21 | .052 | **0.49** |  |
| **Total**  raw score, mean (SD)^d^ | 120.5 (26.2)  n = 33 | 153.6 (29.4)  n = 21 | **<.001** | **1.19** |  |
| **Problem Behavior** |  |  |  |  |  |
| **Child Behavior Checklist** |  |  |  |  |  |
| **Internalizing**  T-score, mean (SD) | 61.6 (9.2)  n = 33 | 52.5 (15.5)  n = 21 | **.006** | **0.71** |  |
| **Externalizing**  T-score, mean (SD) | 54.2 (7.2)  n = 33 | 44.6 (14.0)  n = 21 | **.001** | **0.86** |  |
| **Total Problems**  T-score, mean (SD) | 62.6 (7.6)  n = 33 | 50.5 (14.3)  n = 21 | **<.001** | **1.06** |  |
| **Vineland Adaptive Behavior Scale** |  |  |  |  |  |
| **Communication**  standard score, mean (SD) | 65.6 (19.4)  n = 31 | 94.6 (27.3)  n = 19 | **<.001** | **1.22** |  |
| **Daily Living Skills**  standard score, mean (SD) | 66.0 (14.5)  n = 31 | 96.3 (25.9)  n = 19 | **<.001** | **1.44** |  |
| **Socialization**  standard score, mean (SD) | 65.8 (16.3)  n = 30 | 99.4 (23.6)  n = 19 | **<.001** | **1.66** |  |
| **Motor Skills**  standard score, mean (SD) | 71.1 (16.7)  n = 27 | 86.1 (14.4)  n = 14 | **.013** | **0.96** |  |
| **Adaptive Behavior Composite**  standard score, mean (SD) | 63.0 (15.8)  n = 30 | 97.4 (17.5)  n = 19 | **<.001** | **2.06** |  |
| **Internalizing**  v-scale score, mean (SD) | 20.2 (2.1)  n = 30 | 16.8 (3.3)  n = 18 | **<.001** | **1.23** |  |
| **Externalizing**  v-scale score, mean (SD) | 16.3 (2.4)  n = 30 | 15.0 (2.8)  n = 18 | .092 | **0.51** |  |
|  |  |  |  |  |  |

ASD=autism spectrum disorder; IQ=intelligence quotient; SD=standard deviation

Standard score: mean=100, SD=15; T-score: mean=50, SD=10

Significant p values (<.05) and medium to large effect sizes (Cohen’s d ≥.50) are bolded

^‡^Values reported are least squares mean and standard error, after controlling for age

^a^Higher scores indicate poorer cognitive performance

^b^Raw score interpretation varies slightly with child age. For all age groups, scores <47 raise suspicion for a developmental coordination disorder.

^c^Higher scores indicate more repetitive behavior. Scores should be very low (near the floor) for typically developing children.

^d^Higher scores indicate lower symptom severity. Typical performance score ranges: Tactile Sensitivity 30-35, Taste/Smell Sensitivity 15-20, Movement Sensitivity 13-15, Underresponsive/Seeks Sensation 27-35, Auditory Filtering 23-30, Low Energy/Weak 26-30, Visual/Auditory Sensitivity 19-25, Total 155-190.
