# Supplementary material for: Neurobehavioral phenotype of autism spectrum disorder associated with germline heterozygous mutations in PTEN
Source: Transl Psychiatry. 2019 Oct 8;9:253. doi: 10.1038/s41398-019-0588-1 (PMC6783427; doi:10.1038/s41398-019-0588-1)
Supplement: Supplementary file 5 — Supplemental Table 4 [file 41398_2019_588_MOESM5_ESM.docx]

| **Supplemental Table 4. Group Differences on Cognitive & Behavioral Measures Between *PTEN*-ASD**  **and Macro-ASD** | | | | | | | | | | | | | | | |
| --- | --- | --- | --- | --- | --- | --- | --- | --- | --- | --- | --- | --- | --- | --- | --- |
|  | ***PTEN-ASD***  **n = 36** | | **Macro-ASD**  **n = 25** | | **p** | | **Cohen’s d** | | |  |  |  |  |  |  |
| **Global Cognitive Ability** | | | | | | | | | | | | | | | |
| **Stanford Binet Intelligence Scales** |  | |  | |  | |  | | | | |  |  |  |  |
| **Full Scale IQ**  standard score, mean(SD) | 57.3 (20.1)  n = 33 | | 68.8 (25.3)  n = 26 | | .054 | | **0.50** | | | | |  |  |  |  |
| **Verbal IQ**  standard score, mean(SD) | 58.2 (21.7)  n = 33 | | 67.8 (24.5)  n = 26 | | .118 | | 0.41 | | | | |  |  |  |  |
| **Nonverbal IQ**  standard score, mean(SD) | 58.1 (18.9)  n = 33 | | 72.7 (25.6)  n = 26 | | **.022** | | **0.65** | | | | |  |  |  |  |
| **Attention / Impulsivity** |  | |  | |  | |  | | | | |  |  |  |  |
| **Continuous Performance Test** |  | |  | |  | |  | | | | |  |  |  |  |
| **Omissions**  T-score, mean (SD)^a^ | 80.7 (13.8)  n = 18 | | 68.8 (18.3)  n = 21 | | **.020** | | **0.73** | | | | |  |  |  |  |
| **Commissions**  T-score, mean (SD)^‡,a^ | 69.5 (4.0)  n = 18 | | 62.1 (3.7)  n = 21 | | .185 | | **0.61** | | | | |  |  |  |  |
| **Working Memory** |  | |  | |  | |  | | | | |  |  |  |  |
| **Stanford Binet Intelligence Scales** |  | |  | |  | |  | | | | |  |  |  |  |
| **Working Memory**  standard score, mean (SD) | 62.5 (22.0)  n = 33 | | 69.2 (25.0)  n = 25 | | .271 | | 0.28 | | | | |  |  |  |  |
| **Processing Speed** |  | |  | |  | |  | | | | |  |  |  |  |
| **Wechsler Processing Speed Index**  Standard score, mean (SD) | 60.3 (20.5)  n = 23 | | 70.4 (19.5)  n = 19 | | .100 | | **0.50** | | | | |  |  |  |  |
| **Continuous Performance Test** |  | |  | |  | |  | | | | |  |  |  |  |
| **Hit Reaction Time**  T-score, mean (SD)^a^ | 70.3 (12.2)  n = 10 | | 59.1 (14.1)  n = 17 | | **.034** | | **0.85** | | | | |  |  |  |  |
| **Executive Functions** |  | |  | |  | |  | | | | |  |  |  |  |
| **Behavior Rating Inventory of Executive Function** | | | | | | | | |  | | | | |  |  |
| **Global Executive Composite**  T-score, mean (SD)^a^ | | 66.4 (11.0)  n = 27 | | 70.6 (10.3)  n = 21 | | .265 | | 0.39 | | | | |  |  |  |
| **Behavioral Regulation Index**  T-score, mean (SD)^a^ | | 62.8 (13.0)  n = 21 | | 66.3 (13.1)  n = 19 | | .444 | | 0.27 | | | | |  |  |  |
| **Inhibit**  T-score, mean (SD)^a^ | | 63.9 (13.4)  n = 28 | | 65.1 (15.1)  n = 21 | | .756 | | 0.08 | | | | |  |  |  |
| **Shift**  T-score, mean (SD)^a^ | | 62.1 (12.2)  n = 28 | | 67.3 (13.1)  n = 21 | | .189 | | 0.41 | | | | |  |  |  |
| **Emotional Control**  T-score, mean (SD)^a^ | | 58.9 (12.9)  n = 28 | | 61.1 (13.1)  n = 21 | | .579 | | 0.17 | | | | |  |  |  |
| **Metacognition Index**  T-score, mean (SD)^a^ | | 65.0 (12.1)  n = 22 | | 68.9 (8.9)  n = 19 | | .325 | | 0.37 | | | | |  |  |  |
| **Initiate**  T score, mean (SD)^a^ | | 64.6 (13.4)  n = 21 | | 67.1 (9.7)  n = 19 | | .55 | | 0.21 | | | | |  |  |  |
| **Working Memory**  T-score, mean (SD)^a^ | | 68.9 (15.0)  n = 29 | | 72.3 (10.9)  n = 21 | | .433 | | 0.26 | | | | |  |  |  |
| **Plan/Organize**  T-score, mean (SD)^a^ | | 65.6 (15.9)  n = 28 | | 69.5 (9.2)  n = 21 | | .355 | | 0.26 | | | | |  |  |  |
| **Organization of Materials**  T-score, mean (SD)^a^ | | 54.4 (11.3)  n = 21 | | 56.1 (9.9)  n = 19 | | .647 | | 0.16 | | | | |  |  |  |
| **Monitor**  T-score, med (IQR)^a^ | | 71 (58-76)  n = 20 | | 70 (60-76)  n = 18 | | .958 | | 0.09 | | | | |  |  |  |
| **Language** | |  | |  | |  | |  | | | | |  |  |  |
| **Peabody Picture Vocabulary Test**  standard score, mean (SD) | | 61.1 (32.6)  n = 34 | | 71.8 (32.2)  n = 25 | | .175 | | 0.33 | | | | |  |  |  |
| **Expressive Vocabulary Test**  standard score, mean (SD) | | 60.7 (30.7)  n = 33 | | 72.4 (31.5)  n = 25 | | .128 | | 0.38 | | | | |  |  |  |
| **Visuospatial** | |  | |  | |  | |  | | | | |  |  |  |
| **Visual-Motor Integration**  standard score, mean (SD) | | 63.2 (20.2)  n = 33 | | 71.5 (21.3)  n = 23 | | .124 | | 0.40 | | | | |  |  |  |
| **Motor** | |  | |  | |  | |  | | | | |  |  |  |
| **Developmental Coordination Disorder Questionnaire**  raw score, mean (SD)^b^ | | 26.3 (8.7)  n = 32 | | 33.0 (10.6)  n = 22 | | **.034** | | **0.69** | | | | |  |  |  |
| **Autism Symptoms** | |  | |  | |  | |  | | | | |  |  |  |
| **Autism Diagnostic Observation Schedule**  raw score, med (IQR) | | 7 (5-8)  n = 35 | | 8 (7-9.5)  n = 21 | | **.042** | | **0.56** | | | | |  |  |  |
| **Social Responsiveness Scale**  T-score, med (IQR) | | 76.5 (70-83)  n = 34 | | 74.5 (69-81)  n = 22 | | .940 | | 0.02 | | | | |  |  |  |
| **Repetitive Behavior Scale**  raw score, med (IQR)^c^ | | 21 (12-48)  n = 33 | | 23.5 (17-31)  n = 22 | | .874 | | 0.25 | | | | |  |  |  |
| **Sensory Functioning** | |  | |  | |  | |  | | | | |  |  |  |
| **Short Sensory Profile** | |  | |  | |  | |  | | | | |  |  |  |
| **Tactile Sensitivity**  raw score, med (IQR)^d^ | | 27 (22-31)  n = 33 | | 29 (26-32)  n = 23 | | .331 | | 0.37 | | | | |  |  |  |
| **Taste/Smell Sensitivity**  raw score, med (IQR)^d^ | | 13 (7-20)  n = 33 | | 19 (12-20)  n = 23 | | .241 | | **0.50**^┼^ | | | | |  |  |  |
| **Movement Sensitivity**  raw score, med (IQR)^d^ | | 12 (9-15)  n = 33 | | 13.5 (11-15)  n = 23 | | .522 | | 0.28 | | | | |  |  |  |
| **Under-responsive/Seeks Sensation**  raw score, mean (SD)^d^ | | 20.3 (7.7)  n = 33 | | 23.6 (5.2)  n = 23 | | .079 | | **0.50** | | | | |  |  |  |
| **Auditory Filtering**  raw score, mean (SD)^d^ | | 18.0 (5.1)  n = 33 | | 17.8 (4.1)  n = 23 | | .900 | | 0.04 | | | | |  |  |  |
| **Low Energy/Weak**  raw score, med (IQR)^d^ | | 15 (8-23)  n = 33 | | 22 (12-28)  n = 23 | | .120 | | **0.50**^┼^ | | | | |  |  |  |
| **Visual/Auditory Sensitivity**  raw score, mean (SD)^d^ | | 16.2 (4.8)  n = 33 | | 17.3 (4.2)  n = 23 | | .422 | | 0.24 | | | | |  |  |  |
| **Total**  raw score, mean (SD)^d^ | | 120.5 (26.2)  n = 33 | | 135.8 (23.4)  n = 22 | | **.039** | | **0.62** | | | | |  |  |  |
| **Problem Behavior** | |  | |  | |  | |  | | | | |  |  |  |
| **Child Behavior Checklist** | |  | |  | |  | |  | | | | |  |  |  |
| **Internalizing**  T-score, mean (SD) | | 61.6 (9.2)  n = 33 | | 58.8 (8.6)  n = 21 | | .511 | | 0.31 | | | | |  |  |  |
| **Externalizing**  T-score, mean (SD) | | 54.2 (7.2)  n = 33 | | 53.6 (10.3)  n = 21 | | .703 | | 0.07 | | | | |  |  |  |
| **Total Problems**  T-score, mean (SD) | | 62.6 (7.6)  n = 33 | | 61.3 (7.7)  n = 21 | | .689 | | 0.17 | | | | |  |  |  |
| **Vineland Adaptive Behavior Scale** | |  | |  | |  | |  | | | | |  |  |  |
| **Communication**  standard score, mean (SD) | | 65.6 (19.4)  n = 31 | | 65.3 (18.2)  n = 18 | | .954 | | 0.02 | | | | |  |  |  |
| **Daily Living Skills**  standard score, mean (SD) | | 66.0 (14.5)  n = 31 | | 62.9 (14.3)  n = 18 | | .565 | | 0.22 | | | | |  |  |  |
| **Socialization**  standard score, mean (SD) | | 65.8 (16.3)  n = 30 | | 61.4 (14.9)  n = 18 | | .428 | | 0.28 | | | | |  |  |  |
| **Motor Skills**  standard score, mean (SD) | | 71.1 (16.7)  n = 27 | | 79.0 (22.3)  n = 14 | | .183 | | 0.37 | | | | |  |  |  |
| **Adaptive Behavior Composite**  standard score, mean (SD) | | 63.0 (15.8)  n = 30 | | 61.8 (15.0)  n = 18 | | .745 | | 0.08 | | | | |  |  |  |
| **Internalizing**  v-scale score, mean (SD) | | 20.2 (2.1)  n = 30 | | 19.7 (2.2)  n = 18 | | .505 | | 0.21 | | | | |  |  |  |
| **Externalizing**  v-scale score, mean (SD) | | 16.3 (2.4)  n = 30 | | 17.3 (2.8)  n = 18 | | .204 | | 0.39 | | | | |  |  |  |
|  | |  | |  | |  | |  | | |  | | | |  |

ASD=autism spectrum disorder; IQ=intelligence quotient; SD=standard deviation

Standard score: mean=100, SD=15; T-score: mean=50, SD=10

Significant p values (<.05) and medium to large effect sizes (Cohen’s d ≥.50) are bolded

^‡^Values reported are least squares mean and standard error, after controlling for age

^a^Higher scores indicate poorer cognitive performance

^b^Raw score interpretation varies slightly with child age. For all age groups, scores <47 raise suspicion for a developmental coordination disorder.

^c^Higher scores indicate more repetitive behavior. Scores should be very low (near the floor) for typically developing children.

^d^Higher scores indicate lower symptom severity. Typical performance score ranges: Tactile Sensitivity 30-35, Taste/Smell Sensitivity 15-20, Movement Sensitivity 13-15, Underresponsive/Seeks Sensation 27-35, Auditory Filtering 23-30, Low Energy/Weak 26-30, Visual/Auditory Sensitivity 19-25, Total 155-190.
